# Roadside Unit Deployment in Internet of Vehicles Systems: A Survey

**DOI:** 10.3390/s22093190

**Published:** 2022-04-21

**Authors:** Abderrahim Guerna, Salim Bitam, Carlos T. Calafate

**Affiliations:** 1 Department of Computer Science, Mohamed Boudiaf University of M’sila, M’sila 28000, Algeria; 2 LESIA Laboratory, Department of Computer Science, Mohamed Khider University of Biskra, Biskra 07000, Algeria; s.bitam@univ-biskra.dz; 3 Computer Engineering Department, Universitat Politècnica de València (UPV), 46022 Valencia, Spain

**Keywords:** VANET, Internet of Vehicles (IoV), roadside unit (RSU), static deployment, dynamic deployment

## Abstract

In recent years, the network technology known as Internet of Vehicles (IoV) has been developed to improve road safety and vehicle security, with the goal of servicing the digital demands of car drivers and passengers. However, the highly dynamical network topology that characterizes these networks, and which often leads to discontinuous transmissions, is one of the most significant challenges of IoV. To address this issue, IoV infrastructure-based components known as roadside units (RSU) are designed to play a critical role by providing continuous transmission coverage and permanent connectivity. However, the main challenges that arise when deploying RSUs are balancing IoVs’ performances and total cost so that optimal vehicle service coverage is provided with respect to some target Quality of Service (QoS) such as: service coverage, throughput, low latency, or energy consumption. This paper provides an in-depth survey of RSU deployment in IoV networks, discussing recent research trends in this field, and summarizing of a number of previous papers on the subject. Furthermore, we highlight that two classes of RSU deployment can be found in the literature—static and dynamic—the latter being based on vehicle mobility. A comparison between the existing RSU deployment schemes proposed in existing literature, as well as the various networking metrics, are presented and discussed. Our comparative study confirms that the performance of the different RSU placement solutions heavily depends on several factors such as road shape, particularity of road segments (like accident-prone ones), wireless access methods, mobility model, and vehicles’ distribution over time and space. Besides that, we review the most important RSU placement approaches, highlighting their strengths and limitations. Finally, this survey concludes by presenting some future research directions in this domain.

## 1. Introduction

With the dramatic growth in vehicular traffic and congestion on roadways in recent years, driving is becoming increasingly complex and dangerous. As a result, the global number of automobile accidents and fatalities is growing year after year; therefore, securing traffic becomes not only a necessity, but also an imperative [1]. Consequently, a new research area known as intelligent transportation systems (ITS) [2] is established, in which a specific type of network known as vehicular ad hoc networks (VANETs) is born, which considers every vehicle as a mobile node [3]. VANET is a wireless network that is primarily based on vehicle-to-vehicle (V2V) communication, which assures message transmission when two or more vehicles are in the same transmission range, and even beyond that range through multi-hopping [4]. Current VANETs are insufficient to fulfill future needs due to the use of a pure ad-hoc network architecture, unreliable Internet service, incompatibility with personal devices, non-cooperation with cloud computing, low service accuracy, and operational network dependency [5]. In the last several years, the progressions in 5G network communication [6], and the increasing need for processing information and computational tasks on vehicles, and the rapid development of the Internet of Things (IoT) [7], has caused the traditional paradigm of Vehicle Ad hoc Networks (VANETs) to evolve towards the Internet of Vehicles (IoV) [8]. Due to the heterogeneous nature of IoV, high vehicle speeds, unpredictable density, and traffic environment obstacles or traffic congestion [9], a new vehicular infrastructure was conceived to ensure the sustainability of the vehicles’ communication: roadside units (RSU) [10]. Because of its stable and high communication, computing, and cache capabilities, the Roadside Unit (RSU) is an important component in the IoV [11], which mainly plays a role in collecting and analyzing traffic messages given from smart vehicles, or even providing drivers and passengers with Internet access [12]. Therefore, V2V communication was subsequently improved by adding a new transmission method known as vehicle-to-roadside units (V2R) communication [13]. V2R communications are established if the vehicle is within the transmission range of the RSUs. This way, the messages are delivered directly to the RSU. Conversely, when the vehicle is out side the RSU transmission area, the connectivity is defined through a multi-hop relaying. Despite their numerous advantages, the presence of RSUs is expected to be reduced due to the high deployment, energy constraints and maintenance costs, particularly when deployed on a large-scale [14]. To address the challenges associated with RSU deployment, determining the ideal locations in a particular region under the cost constraints so as to maximize network performances becomes an essential issue. RSUs placement is, therefore, described as the process of determining the optimum combination of RSUs on candidate places based on given conditions in order to achieve the specified requirements (e.g., best connectivity, coverage, low deployment cost). Finding the best RSU deployment is an NP-Hard combinatorial optimization issue [15]. In fact, there have been a large number of research works focusing on the RSU deployment optimization issues in vehicular environments. To the best of our knowledge, two survey papers [16,17] addressed the RSU deployment (RD) issues. In [16], the authors cover the topic of infrastructure-based vehicular networks, with one section discussing RSU deployment—while Ackels et al. [17] fussed over four aspects of RSU deployment: formulations, solutions, cost functions, and simulations. However, they do not propose a taxonomy of RSU deployment approaches. 

This paper provides a review and classification of different RSU deployment approaches in Vehicular Networking. Based on the mobility of vehicles, and strategy for placing RSUs in geographic areas, we propose to classify the reviewed studies into two categories, namely schemes based on static deployment, and schemes based on dynamic deployment. In the static deployment, RSUs are deployed at fixed places on the studied geographical area. This category could be divided into five sub-classes according to the strategy and models used for the RSUs deployment. These five sub-classes are enumerated as follows:

1. RSU deployment based on analytic study;
2. Geometry coverage model;
3. Transmission time strategy;
4. Maximum coverage model;
5. Network density approach.

According to the type and function of the wireless devices adopted by vehicles, the dynamic deployment class can be separated into four sub-categories as follows:

1. Vehicle used as temporary RSU;
2. Parked cars can be used as RSUs;
3. Similarly to buses of regular lines being used as RSUs;
4. Unmanned aerial vehicles (UAV) act as RSUs.

In this survey, the most important RSU placement approaches are reviewed, highlighting their strengths and limitations. The main purpose of this paper is to help the research community to identify alternative solutions, and select the appropriate strategies to place RSUs in vehicular networks in an optimal manner.

The rest of the paper is organized as follows: Section 2 describes the VANET background. Section 3 explains the deployment of roadside units in a VANET, the primary optimization constraints, and the most important optimization metrics. In Section 4, we present different RSU deployment approaches, and we classify them into two categories: static and dynamic deployment categories. This also includes some performance criteria of each deployment class. Section 5 presents open research directions to improve the efficiency of the RSU deployment. Finally, Section 6 concludes the paper. For ease of reading,

## 2. Vehicular Networking: Definition and Deployment

**Figure 1:**
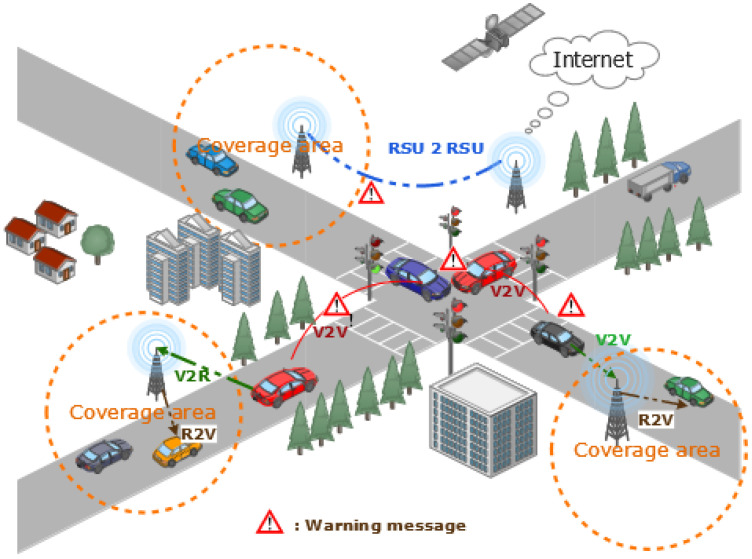
Communication modes in VANETs.

VANET is a subtype of Mobile Ad-hoc Network (MANET), where mobile nodes are smart vehicles able to create a spontaneous (ad hoc) network in order to transmit data packets between them [18]. Such vehicles are comprised of On Board Units (OBU) for computing and transmitting messages, GPS (Global Positioning System) for location detection, EDR (Event Data Recorder), and sensors (radar and ladar) [19]. This equipment is used to sense traffic congestion and avoid any serious traffic accident; in addition, they relay this information through the various communication modes available in the scope of a vehicular network environment, as shown in Figure 1. A VANET is also formed by a collaboration between vehicles and fixed infrastructures called roadside units (RSUs) [20], which help with data transmission. RSU is a fixed device along roads that is equipped with at least a network device for short-range wireless communications based on IEEE 802.11p [21], with 75 MHz of a Dedicated Short Range Communication (DSRC) spectrum at 5.9 GHz [22]. Within their coverage area, an RSU plays an important role for collecting and analyzing traffic data generated by smart vehicles. Additionally, RSUs can serve as a gateway to other communication networks, such as the Internet [23].

In VANETs, there are several communication mechanisms available, including the vehicle to-vehicle (V2V) mode, which is a pure ad-hoc communication without fixed infrastructure, vehicle-to-roadside units (V2R), or roadside units-to-vehicle (R2V) communications, which allows a vehicle to communicate with road side units primarily for collecting information and analyzing traffic data, and even a hybrid communication mode [2,19]. The latter is a combination between V2V and V2R communications, whereby a vehicle can directly communicate with the road infrastructure; in addition, a vehicle can communicate via multi-hopping with other vehicles when direct transmission to an RSU is not possible with a single hop [3]. We should note that an RSU can directly transmit data to another RSU in roadside unit-to-roadside unit (RSU 2 RSU) communication to facilitate computation, network load-balancing, and information sharing [24]. Figure 1 shows a VANET architecture with different transmission modes.

VANET are essentially characterized by a high mobility of vehicles, leading to a highly dynamic network topology because cars driving at different speeds can result in frequent fragmentation in their network connectivity [25].

Many VANET-based applications have been developed, which may be divided into three categories: safety, efficiency and comfort applications [26]. The goal of safety applications is to reduce the frequency of traffic accidents, whereas efficiency applications provide information and recommendations for traffic optimisation. Finally, comfort applications are designed to meet digital needs of drivers and passengers, such as accessing the Internet, locating the nearest restaurant, hotel, or gas station.

## 3. Deployment of Roadside Units in VANET: An Overview

Since VANETs have to face some of the critical tasks of ad hoc networks, such as dynamic topology, high vehicle speeds that moving at different directions, short life of connectivity, etc., V2V communication may have poor performance in the collection and transmission of the data provided by vehicles. This complicates the development of delay-sensitive applications on VANETs even further. To meet these needs, deploying a vehicular infrastructure (RSU) is a key solution to improving message dissemination performance in the VANET [27]. RSU placement is described as the procedure of determining the optimal combination of RSUs in a certain target area according to the given parameters so as to achieve the specified requirements (e.g., best connectivity [28,29], maximum coverage [30], low deployment cost). In this part, we will look at how to place RSUs in a researched region to obtain the optimum network performance. In this section, we first describe the problem of RSU deployment, and then detail the objectives that have been addressed in order to achieve the best network performance.

### 3.1. Problem Statement

Due to the high cost of deploying and maintaining RSUs, a significant challenge is determining how to deploy a small number of RSUs while ensuring excellent network performance. To ensure such performance, VANETs pose several challenges for RSU deployment in terms of coverage, network connectivity, data dissemination, packet routing, security, privacy, and so on [31]. However, coverage is one of the key performance metrics used to assess the quality of service (QoS) supplied in a network. In other words, the optimization’s main purpose is to find a compromise between network coverage and cost. RSU deployment is modeled as a constrained optimization problem with multiple objectives such as improving network coverage, optimizing network connection, and decreasing RSU deployment costs. In a geographical area, we can usually find various feasible subsets of locales for deploying RSUs. If there were 100 candidate places and 10 RSU to be deployed, there would be $1.73\times 10^{13}$ possible placements [32]. This RSU deployment problem is considered as a combinatorial optimization problem [33], and has also been proved to be NP-hard [15].

### 3.2. Tackled Objectives in RSU Deployment

The majority of proposed RSU placement methods in the literature have focused on the aims of increasing transmission coverage and achieving good network connection. The transmission coverage of the monitored area can be ensured by careful planning of the vehicle densities on the concerned traffic, while achieving a strongly connected network topology. Generally, the RSU deployment mainly includes the following performance factors.

- *Maximizing the transmission coverage area:* An area is considered as covered by a RSU if it remains within its transmission range. Due to the RSU’s short communication range, a dense deployment of RSUs is required to achieve ubiquitous coverage throughout a city; nevertheless, service providers may be forced to charge high RSU access fees, discouraging consumers from using the service [34,35]. The RSU coverage allows for answering the question: for how long are the vehicles able to detect an RSU? Additionally, transmission coverage formulations can try to find the best location in the physical space with the goal of having at least one RSU within a transmission range.
- *Network connectivity*: because of its dynamic nature, a VANET frequently experiences intermittent connectivity, which increases the delay in disseminating the gathered road conditions’ information, and hence affects the quality of service (QoS) provided to users [36]. To solve this issue, the roadside units (RSUs) can be deployed as an aid for the VANET to increase network connectivity, reduce transmission delays, and improve communication ranges [37]. If the communication range of an RSU exceeds the communication range of a vehicle, the connectivity analysis remains unaffected [38].
- *Cost deployment minimization*: The deployment of RSUs in a road network necessitates investment and maintenance. For example, if RSUs are widely deployed around the city, coverage will be expanded, but the RSU setup cost may be too high (between $13,000 and $15,000 per unit capital cost, and up to $2400 per unit per year for operation and maintenance [39]. Hence, many large RSU deployment strategies might fail not just because of high initial setup costs, but also because of little used RSU waste energy. To address this issue, finding the optimal balance between sleep or active mode for RSUs is a primary strategy to minimize its overall energy consumption while maintaining network connectivity [40].

As a result, to achieve optimum performance in terms of transmission range, network connectivity, and QoS, solutions must ideally plan RSU deployment following a strict budget in a given region [41,42]. Therefore, the RSU deployment should be optimized depending on various factors such as traffic patterns and vehicle density, variety of services that appear, and a communication profile, as well as the technical effectiveness at achieving the limits of the underlying communication mechanisms [43].

### 3.3. Problem Modelling

In the literature, various RSU deployment strategies have been used that aim at improving the transmission coverage in vehicular networking, which transformed the deployment problem into the classic Coimbatore’s optimization problems such as: Integer Linear Programming (ILP) [44,45] Set coverage problem (SCP) [46], maximum coverage problem (MCP) [47,48], Knapsack problem [47,49], facility location problem (FLP) [50,51], vertex coverage (VCP) problem [52,53], and Budgeted Maximum Coverage Problem (BMCP) [54]. To summarize, we provide a qualitative overview of static and dynamic deployment approaches. We have also included a column called “model” detailing some approaches that adopted this method.

### 3.4. Performance Metrics for RSU Deployment

Due to VANET characteristics such as node mobility, frequent topology changes, heterogeneous and unbounded environments, and a vehicle’s limited transmission radius, broadcasting in VANETs is a difficult task. However, safety messages are time-sensitive and have specific performance and QoS requirements. QoS is defined as the set of requirements that the network must meet during the packet transmission flow from source to destination [55]. Many parameters can be considered when measuring QoS:

- *Coverage ratio:* This important metric is calculated by dividing the number of valid coverage sub-roads by the total number of sub-roads in the road network; it indicates the ratio of road segments coverage in the network [56]. Subtracting duplicated sub-roads from all sub-roads yields the number of legitimate coverage sub-roads.
- *Overlapping coverage area:* Large coverage areas that overlap with nearby RSUs waste resources and reduce the capacity to disseminate information over larger regions [57]. In addition, such RSUs may deal with some redundant duplicated traffic messages generated by vehicles within the overlapped area covered by more than one RSU. As a result, every RSU deployment strategy must consider reducing the extent of the overlapping coverage of RSUs to the bare minimum.
- *Packet delay:* The packet delay is a primary metric to guarantee the quality of service for VANET [58]. It is not only important to receive the packet, but to receive it within the maximum eligible delay as well. Any packet received after this time limit hinders service availability.
- *Packet loss ratio:* Packet loss refers to the number of packets dropped in transmissions, which is used to measure the ability of a network to relay. This measure is based on the maximum allowable delay, and any packet received after this limit is considered as lost [43]. By subtracting the number of packets successfully broadcast during the delay from all packets in the deployed region, the number of packets lost is calculated [15].
- *Packet delivery rates:* The packet delivery rate is derived by dividing the total number of packets received by the target RSUs by the total number of packets coming from vehicles. It measures the percentage of the transmitted data packets that are successfully received [59].

## 4. Taxonomy of RSU Deployment

**Figure 2:**
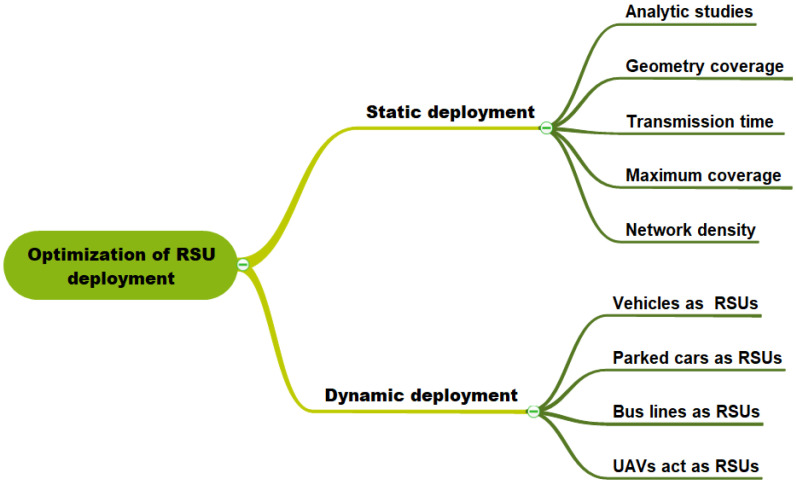
Taxonomy of RSU deployment.

In the research community, an extensive number of papers have been conducted to develop efficient strategies integrating network coverage and low-cost RSU deployment. Depending on the coverage objectives and deployment cost, we categorize the RSU deployment techniques into two categories: static deployment and dynamic deployment, as shown in Figure 2.

In the static deployment, the RSUs are deployed in a static location on the target geographic areas to improve network coverage towards moving vehicles. On the contrary, dynamic deployment is based on the idea of using some vehicles equipped with an on-board computer and wireless communication device to be used as RSUs. Both static and dynamic VANET RSU deployment strategies are discussed in the next sections.

### 4.1. Static Deployment

For simplicity, many deployment studies usually assume that RSUs are deployed at fixed locations in the road network. In this section, we will look at each of the different research studies, and we propose to classify them into five sub-classes according to their models and deployment objectives in the road network, as shown in Figure 2. For each study, we describe the proposed model, and the main idea proposed as an optimal solution for RSU deployment in VANET. Moreover, we perform a qualitative comparison between the different strategies of static RSU deployment.

#### 4.1.1. RSU Deployment Based on Analytic Studies

In this sub-class, the RSU deployment issue is based on analysis and mathematical proof to assure the correctness of proposed approaches. Liya et al. [60] proposed the randomized procedure for estimating an approximate optimal distance *d* for deploying the RSUs on the highway such that a security message may be broadcast to RSUs from all accident zones in time *t*, with at least a particular probability parameter *p*. This distance is estimated by gradually approaching the ideal distance from an initial distance until the VANET is unable to satisfy the connection requirements. The initial distance is estimated as $d_{0}=2R_{0}$, where $R_{0}$ is the maximum distance for wireless transmission from one vehicle or one RSU to another vehicle or to another RSU. In this proposal, the authors presented a mathematical proof of the correctness of their algorithm. As a critical examination, the approach described in the study appears to be highly promising for large-scale deployments, and we may be able to increase the technique’s efficiency by employing a better strategy for allocating RSUs. Therefore, it is better to explore the wireless mesh backbones, whose placement of RSUs could by ensured by wireless interconnection.

In [44], the authors proposed a Capacity Maximization Placement (CMP) schema to deploy a minimum number of RSUs. This technique is applied in a highway, such that the achievable aggregate throughput in the network can be maximized. In order to allow a vehicle to access RSUs, two scenarios are taken into account; either a direct access to a RSU, when the vehicle is in range transmission of this RSU, or using multi-hop relay if this vehicle is between two RSUs. The hotspots are found by dividing the zone in question into fixed size cells and assigning a coverage value to each cell based on geometrical factors such as wireless interference, vehicle population distribution, and vehicle speed. An integer linear programming (ILP) model is employed to define this problem such that the total flow in the network could be maximized. This work considered the influence of wireless interference, vehicle population distribution, and vehicle speeds in the problem formulation. The results obtained showed that the CMP method surpasses the other two placement strategies, namely, uniformly distribution and hotspot placement, in terms of the aggregate throughput and the deployment budget, the number of RSUs required. However, the problem formulation is purely analytic, not implemented by any algorithm and simulation.

Aslam et al. [45] implemented two optimization schemes for solving the RSU deployment problem: an analytical method known as Binary Integer Programming (BIP), and a new strategy known as Balloon Expansion Heuristic (BEH). The objective of the methods was to reduce average reporting time, meanwhile ensuring a certain coverage area and a fixed number of RSUs in an urban environment. BIP utilizes the branch and bound approach to find an optimal analytical solution, whereas the BEH heuristic inspires the balloon expansion analogy to find the best solution. In BEH, an RSU coverage area is regarded as a balloon that dilates gradually in the two-dimensional space until the desired percentage of the area covered, under the average reporting time constraint, is obtained. Simulation results confirm that the BEH procedure outperforms the BIP method in terms of the computational cost and scalability. However, this research approach remains simple and did not take into account realistic topologies that include road complexity and obstacles.

In most cases, RSU Deployment (RD) models in the literature lack the capacity to depict curve shapedroads and non-uniform statistics on roads. Based on taking into account these characteristics, Gao et al. [61,62] suggest researching into the RSU deployment considering connectivity in the one-dimensional context, where the road network is considered as one straight line. The authors looked at the one-dimensional RSU deployment problem (D1RD), which involves deploying *n* RSUs with varying coverage radius under constraints. Given that the search space for the D1RD issue involving several RSUs is enormous in size, the authors analysed the attributes of the optimal solutions to the D1RD problem with a single RSU, and then extended it to multiple RSUs in order to reduce the size of the search space. Next, they have proposed a set of approximate algorithms to solve the D1RD problem.

In [61], the authors proposed the Greedy2P3 and Greedy2P3E algorithms to solve the D1RD problem with *n* RSUs of different coverage radii. Since the results showed that the approximate ratio of the Greedy2P3 and Greedy2P3E algorithms is at least $1-{\left(\frac{n-1}{n}\right)}^{2}$, an OptGreDyn approach is developed; it is an optimum solution that combines the greedy and dynamic programming methods. Compared to various existing algorithms, the OptGreDyn approach provides better results in terms of approximate ratio, but better approximation techniques are required for the new RD issue with several heterogeneous RSUs.

The authors in [62] analysed the attributes of the optimal solutions to the D1RD problem with non-uniform profit density. Then, an efficient approach called Dynamic Limiting (DynLim) was proposed, in which the size of the solution search space is reduced significantly by dynamically modifying search space bounds. Furthermore, based on the DynLim approach, an optimal algorithm named OptDynLim is suggested, and its optimally is demonstrated. Compared to the existing algorithms, the simulation results demonstrated that DynLim can minimize the size of the solution search space by more than 99% in most scenarios. As advantages, the proposed model was validated based on strong theatrical analysis and compared to various existing algorithms. Nerveless, the experimental simulations are tested without any real topology area, they do not consider the network performance metrics, and do not take account the mobility traces.

#### 4.1.2. RSUS Deployment Based on Geometry Coverage Model

In this category, each RSU coverage area is considered as a logical coverage area based on the geometry properties. Specifically, logical coverage areas are dynamically expanding in a two-dimensional space.

**Figure 3:**
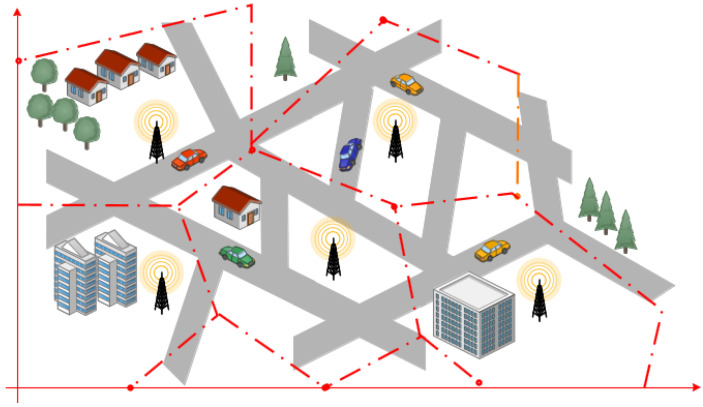
Voronoi diagram approach for RSU deployment in an urban region.

Patil and Gokhale [43] proposed a Voronoï [63] diagram-based algorithm for the effective RSU deployment taking packet loss and delay into account as criteria. Based on the delay threshold of a packet broadcast between two RSUs, the extensive range of RSU defines the contours of the polygon. Any packet delays exceeding this threshold hinder service availability. Figure 3 shows the process of the Voronoï diagram, which produces a set of points in convex polygons according to the geographical area, being divided into convex cells. Furthermore, the resulting map of RSUs shows that there are highly probable areas of overlapping between RSUs. To remove overlapping areas and unattended areas, the extended ranges of any two RSUs in a pair overlap are considered neighbors. Many factors, such as traffic density and junction priority, have little effect on this approach. However, the deployment locations determined by this strategy are not always feasible for installing RSUs, as the placement area did not take into account the private land or obstructions like rivers and buildings.

In urban areas, Cheng and all [64] suggested GeoCover, a geometry-based sparse coverage protocol that investigates the issues of geometrical features of road networks, mobility patterns, and resource limitations. By recognizing hotspots from trace data, GeoCover is capable of depicting mobility patterns and selecting the most valuable road area to be covered. In order to fit the geometrical characteristics, the candidate deployment area is determined using a buffering process based on the road segments’ characteristics. To fulfill budget and quality criteria, the sparse coverage takes into account two variants: Qualified Sparse Coverage (QSC) and Budgeted Sparse Coverage (BSC). To maximize the quality of coverage while respecting the cost under budget constraints, the authors suggest two algorithms: GeoCover-genetic and greedy (Greedy Cover) algorithm. This approach provided good coverage with a reasonable delay and scalability as well. It deployed the RSUs in a hotspot area where the majority of the vehicles are congregated. However, in the real world, if the hotspot area changes for whatever reason, the RSUs must deploy according to the new hotspot discovery procedure. Additionally, the authors failed to examine the global coverage attained by their strategy.

**Figure 4:**
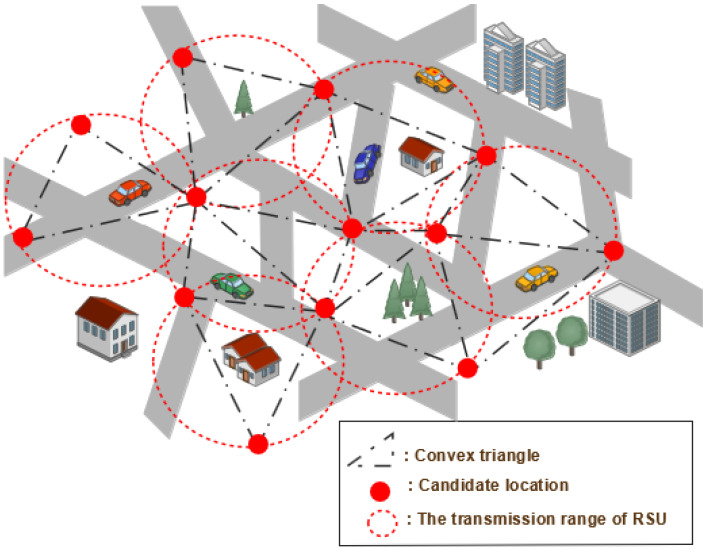
Constrained Delaunay triangulation approach.

In [65], Ghorai and Banerjee modeled the Constrained Delaunay Triangulation (CDT) strategy [66] to optimize the RSU deployment. As a result, the topological region is divided into several convex triangles, the vertices of which designate the RSU candidate location, such that no other RSUs are inside any triangle’s circumcircle (see Figure 4).

The first target of the proposed approach is to place the RSUs in that obstructed area of an urban topology to attain extensive coverage. Then, an optimization strategy was included to get the optimal RSU location and decrease the communication delay in V2R scenarios. According to simulation findings conducted with varied scenario maps, different numbers of RSUs, different vehicle densities, and different vehicle flows showed that the suggested approach gives good results in terms of packet delivery rate, packet loss, and end-to-end latency compared to the GeoCover algorithm and α-coverage algorithm [64] methods. However, this strategy only provides better results when the scenario tested has a simple map with fewer obstacles than a medium or complex map.

Fogue et al. [67] studied the delay-bounded and cost-limited RSU deployment (DBCL) problem in urban VANETs, and transformed the DBCL problem into a variation of the 0–1 Knapsack problem, and a binary differential evolution scheme is proposed to solve it; in particular, it uses a genetic algorithm to increase vehicular communication capabilities and to minimize warning notification time, which is the time required to transmit warning messages to the emergency authorities. This study focuses on how to reduce deployment costs by allocating the exact number of RSUs required to provide the appropriate coverage, as well as to improve the communication capabilities of the vehicles in terms of reduced warning notification time. The study’s findings indicated that DBCL has the capacity to reduce warning notification time, as well as improve vehicular communication capacities in a variety of density and complexity layouts. However, other parameters such as cost of installation and accident information are not incorporated in this design.

#### 4.1.3. RSUs Deployment Based on Transmission Time

In order to transmit delay-sensitive data, the RSU allocation method must be able to collect all traffic data and broadcast it in real-time throughout the network. Liu et al. in [68] studied the delay of transmitting alert messages along a highway in order to ensure that the alert messages can be transmitted to the nearest RSU within a given delay bound. It is evident that reducing critical delay is to provide an emergency answer in a timely manner. In particular, they provided an analytical approach for analyzing the delay in VANETs with fixed transmission distance. Furthermore, the problem is formulated as a coverage problem because the goal is to cover the roads with RSUs in such a way that emergency notifications are sent to RSUs within the set delay constraint. The vehicles are then divided into clusters, with cluster members communicating with one another in no more than two hops. Messages should be carried by vehicles until they meet an RSU if the vehicle clusters are disconnected. The authors developed a genetic algorithm combined with greedy methods to tackle this problem. The simulation results indicated that the solutions are efficient and have a lower time complexity than the existing techniques (greedy and genetic approaches). The relationship between major system factors such as traffic flow density, transmission range, and latency is deduced in this proposal. However, the approach is tested on only one topology.

In [69], the problem addressed is how to deploy a number of *k* RSUs on a highway-like roadway, to maximize network coverage while minimizing the time required for data transmission. This problem is modeled as a Maximum Coverage with Time Threshold Problem (MCTTP), and a genetic algorithm is proposed to solve it. This Time is defined as the minimum time required for a vehicle to contact a RSU and success fully transmits information. The effectiveness of this algorithm depends on two components: fitness and the population initialization process. Fitness is defined as the percentage of covered vehicles in the area. Furthermore, four variants of population initialization are provided: the initialization is completely random, the greedy solution is integrated into the initial random population, the population is half random and half initialized by a modified version of the greedy approach, and the three previous variations are combined. The test results demonstrated that the population initialized by a combination of the greedy method and random initialization outperforms the greedy algorithm (GA). This GA-based technique focuses on V2R communication and does not take cooperative V2V communications into account. Furthermore, the simulation results did not identify the impact on the QoS metrics.

Jalooli et al. [70] introduced the Safety-Based Disconnected RSU Placement process (S-BRP), which is aimed at minimizing the dissemination time for VANETs’ safety applications in multi-hop broadcast schemes. They assume that road intersections have a high probability of accidents, and they propose to place the RSUs at the intersections. Furthermore, their study considers deployment at road segments where the length of the section exceeds the transmission range. Since RSUs are put autonomously without any roadside unit-to-roadside unit (RSU 2 RSU) connection, the RSU installed at a road segment serves as a relay between vehicles. According to this assumption, the absence of (RSU 2 RSU) communication can make the process of deployment very expensive. Hence, this approach needs to find a trade-off between the cost of deploying standalone RSUs and the average dissemination delay.

Ahmed et al. [71] formulated a RSUs placement as a Delay Minimization Problem (DMP) based on Integer Linear Programming (ILP), whose objective is to minimize network latency while staying within the deployment’s total budget on the highway-like roadway scenario. Compared to uniform distribution and cost-effective strategies, the proposed model gives good results in terms of network latency. However, this approach is compared with a simple strategy of deployment that does not consider vehicles’ mobility. In addition, a few more algorithms can be tested and compared.

Yang et al. [15] studied the delay-bounded and cost-limited RSU deployment in urban areas. The Binary Differential Evolution-based Optimal RSU deployment approach (BDERD) is proposed to find the best location of RSUs under the constraints of delay and budget. They demonstrate that it is an NP-hard problem, and suggest a binary differential evolution method to optimize the number of roads covered by deploying RSUs. To begin the first generation, opposite-based learning is used, and a binary differential mutation operator is formed to generate binary coding. To augment population diversity, a random variable is incorporated into the standard crossover operator. In addition, a greedy-based individual reparation and promotion algorithm is adopted to repair infeasible solutions that do not satisfy the constraints imposed. Furthermore, following selection, a solution promotion algorithm is run to promote the best solution discovered during generation. The simulation results showed that BDERD has a greater road coverage ratio and lower packet loss than other schemes. However, this not investigated quality-of-service (QoS)-guaranteed RSU deployment in VANETs.

Ni et al. [50] define the RSU deployment problem in two dimensional (2D) Internet of Vehicles (IoV) networks as a utility-based maximizing problem with an irregular service area for the RSUs. The cost function for their problem is based on the predicted delivery delay requirements, the number of vehicles that may be served concurrently, the benefit of sending the message, and lastly the installation cost. The authors consider the idea of a Facility Location Problem (FLP) to solve the RSU deployment problem, then tackle the problem using a clustering algorithm based on Integer Linear Programming (ILP), and finally formulate the difference between a proposed solution and the optimal solution to the problem. Finally, the proposed strategy is evaluated in comparison to a greedy algorithm, an ILP-based algorithm, and a total deployment algorithm. The results demonstrate the effectiveness and superiority of the proposed solution for IoV network service guarantees over other approaches. This approach, however, is not appropriate for low vehicle density since the transmission delay increases owing to multi-hop communication between the vehicles.

Silva et al. [72] considered that the optimal streaming transmission requires knowing the share of vehicles that periodically contact the RSUs in time intervals smaller than a given threshold. Based on the share of vehicles and time threshold, they proposed the gamma deployment approach defining the location and number of roadside units required for providing the specific coverage. To evaluate this approach, the gamma-related deployment strategy is proposed to ensure that a certain percentage of vehicles consuming data streams passes through small islands of coverage provided by roadside units on a regular basis in order to obtain additional data until reaching the next island of coverage. The gamma-related deployment strategy is then extended to take into account the data transfer rate at which vehicles receive data from roadside units, as well as the data consumption rate of streaming within vehicles, yielding the Gamma-Reload deployment approach. Compared to the RSU deployment strategy based on road density, the proposed approach may achieve significant economy on the costs for setting up the communication network, while providing similar QoS. However, this work did not consider multi-hop communications to measure the vehicles connectivity (i.e., how can we evaluate the connection supplied by the infrastructure-based network, and which sites must be covered first in order to improve network performance when a certain aim is in mind?). In addition, the assumption of Gamma-Reload did not extend to vehicle-to-vehicle communication (i.e., it did not rely on disseminating data, requiring less performance from the infrastructure).

#### 4.1.4. RSU Deployment Based on Maximum Coverage Model

The RUS placement issue is transformed into a Maximum Coverage Problem (MCP) [54] to maximize the number of vehicles that get in contact with the RSUs over the considered area. Definition 1 provides more details for this formulation.

**Definition** **1.** *Suppose a collection of sets $S=\{S_{1},S_{2},\ldots ,S_{n}\}$ defined over a domain of elements $V=\{V_{1},V_{2},\ldots ,V_{m}\}$. Sets may share elements. The goal is to find a k collection of sets $S^{\prime }\subseteq S$ such that the number of covered elements ∣${⋃}_{\left(S_{i}\in S^{\prime }\right)}$∣ is maximized [47].*

The majority of studies such as in [73,74] denote S as the candidate sites for where a VANET infrastructure could be placed, $S^{\prime }$ as the locations set when the RSUs have been installed, and *V* as the number of vehicles covered by *k* RSUs.

Jo and Jeong in [73] described the Greedy Set-Coverage technique, which aims to reduce the number of RSUs while maintaining the required Quality-of-service (QoS) to vehicles in terms of delivery delay. The main idea is to use vehicular traffic statistics to select the best candidate intersections for RSU deployment in order to reduce packet delivery delay. Hence, the deployment problem is modeled as a Set Covering Problem (SCP) to maximize network connectivity, and reduce the probability and time period of network partition with a limited number of RSUs. This approach has the benefit of taking into account both road traffic and data transmission Quality of Service (QoS) in a multi-hop situation. Nonetheless, the findings revealed that the Greedy Set-Cover method does not always outperform uniform placement. In addition, the Greedy Set-Cover computes a minimal number of intersections but does not select the optimal positions of that number of intersections.

Silva et al. [75] developed an RSU deployment strategy based on the partial mobility information, modeling the problem as a probabilistic maximum coverage problem with the aim of finding the places that will cover the most vehicles with at least one RSU. They use the model of urban cells to explain how to generalize the application of migration ratios to big cities. Moreover, they employ the migration rates between adjacent urban cells to split the metropolis into a grid-like form, and identify the specific number of locations that maximize the degree of V2R contact opportunity. The simulation results show that the optimal RSU placement based on the partial mobility information model is better than the full mobility information. Nevertheless, the proposed strategy did not assure that the whole region was covered. They also failed to account for the overall number of RSUs necessary to cover the entire area.

Moura et al. [76] developed the maximum coverage problem with a time threshold that treats the network as a graph while considering the road intersection as a location candidate for deploying RSUs. A genetic algorithm is offered to solve the modeled problem, and a pre-processing procedure based on the betweeness centrality metric is employed to minimize its convergence time. To validate this approach, five different mobility traces from different cities are used in simulation. Compared to a greedy algorithm, the results showed that this coverage and connectivity have immensely improved. However, other parameters such as cost of installation, accident information, and urban impediments are not incorporated in this scheme.

Guerna and Bitam [53] formulated the RSU deployment problem as a multi-objective optimization problem, and, as a result, they suggested a novel genetic intersection-coverage algorithm (GICA) based on the priority notion. This model concentrates on prominent junctions in terms of RSU installation, with the goal of maximizing RSU coverage while lowering interference rate and RSU cost. The results of the experiments show that GICA outperforms the greedy strategy, although it does not account for average connectivity and deployment budget volatility.

To overcome this limitation, they enhanced their previous work developing a new bio-inspired RSU placement system called an ant colony optimization system for RSU deployment in VANET (AC-RDV) [77], where the problem is formulated as a Vertex Coverage Problem (VCP) through a graph-based modeling. AC-RDV is based on the concept of deploying RSUs at high-traffic intersections. As a result, any RSU deployed at each intersection can cover a subset of intersections if these intersections are within the RSUs’ transmission range. Following that, all intersections within this RSU’s transmission range are removed from the deployment candidate set of intersections. The performance of the AC-RDV approach was examined in terms of the number of RSUs deployed, average area coverage, average connectivity, and overlapping ratio. The results showed that the suggested scheme outperformed the typical RSU placement scheme based on the greedy approach (GA) [78], genetic intersection coverage (GICA) [53], and heuristic genetic algorithm (HGA). However, this proposal did not use any realistic urban topology.

Wang et al. [79] proposed a multi-objective differential evolution with a discrete elitist guide (MODE-deg) to deploy the RSUs at traffic light intersections. This process is started by the establishment of a static model in order to conquer the complexity of urban RSU deployment, and the sigmoid function is applied to discrete individual values in the population. Consequently, the individuals that formed the population are selected according to the crowding distance sorting and the fast non-dominated sorting. Next, mutation, crossover and elitist selection are applied to obtain a new generation. In comparison to previous multi-objective evolutionary algorithms, experimental results suggest that MODE-deg can create the best non-dominant solution set with good convergence and diversity. However, this framework does not improve the communication success rate of mobile vehicles in the coverage area.

In [80], the authors proposed a more realistic model called the powerful RSU Deployment Problem Model (RDPM), as a road-network model and a profit model, with the objective of maximizing profit while minimizing the number of RSUs. The number of completely and partially covered road segments determines the profit for this implementation. A genetic algorithm was proposed for solving the RDPM problem. Using simulation, the authors compared their results to those of another approach known as BEH [45]. The RDPM road-network model supports the complex road geometries while also taking into account crucial influencing elements such as the number of lanes and traffic statistics. However, genetic algorithms usually lead to approximate solutions, whereas they provide little insights into the new RD problem. Furthermore, this work does not take into consideration the RSU-deployment-incurred costs.

#### 4.1.5. RSU Deployment Based on Network Density

RSU deployment based on network density considers the density when searching for potential parameter locations for deploying RSUs. Furthermore, the network coverage is greater at a placement with dense traffic than at locations with light traffic.

Locher et al. [32] presented the RSU deployment approach according to a landmark-based aggregation scheme for economy travel time data in road networks. In addition, the landmark-based aggregation system disseminates information about travel time between important points and landmark locations in order to determine how much time a specific active RSU location vector may save. Cars crossing a road segment can carry out an observation of the current travel time between two neighboring landmarks. To identify the optimal placement of supporting units (SUs), an estimate of travel time data is employed as fitness indicators in genetic algorithms. The main advantage of this approach is that it minimizes the required overall bandwidth via a specific aggregation scheme. However, the deployment system concentrated on information aggregation instead of data dissemination. In addition, in real traffic, the vehicles’ movements are unpredictable due to both human and environmental factors.

Chi et al. [78] introduced the intersection priority concept to preferably place RSUs at important intersections. Since network coverage becomes optimal at intersections, RSU deployment based on intersections considers them as candidate RSU deployment locations. Consequently, network coverage is better at a densely trafficked intersection than at a light-trafficked intersection. Three algorithms are presented to serve this purpose: greedy, dynamic, and hybrid algorithms. The greedy algorithm provides a priority list in descending order, and starts by placing the first RSU at the highest intersection priority; then, the intersection located within the transmission range of the RSU is excluded from the priority list. Despite its simplicity, this algorithm can lead to a situation where different RSU transmission ranges will be unnecessarily overlapped. To limit the size of the overlapping region, the dynamic algorithm focuses on ensuring an equitable distribution of RSUs. Finally, the hybrid method combines greedy and dynamic algorithms to distribute RSUs as evenly as possible while maintaining the intersection priority order. The simulation results of this study showed that the dynamic approach provides the best performance, while the hybrid approach produces a middle level of performance between the greedy and the dynamic approaches. As a limitation, this work did not consider the impact of network connectivity on RSU deployment schemes.

Based on the simulation of the urban environment, Barrachina et al. [81] developed a density-based RSU deployment (D-RSU) strategy for delivering emergency-alerting services with the lowest feasible cost in the event of an accident. In urban areas, sites with a high density of vehicles are usually important; consequently, more RSUs should be deployed in these areas. This approach aims to place the RSU in an inverse proportion to the expected density. The authors concluded that, by deploying RSUs in this manner, a uniform coverage area may be achieved, regardless of considerations like traffic density or road network topology. Most importantly, this deployment approach prevents RSUs from being consolidated in a single location, but the cost of deploying RSUs according to the uniform Mesh deployment policy is expensive. In addition, this study does not take into account the street structure of each region when determining the best position for the available RSUs.

Sankaranarayanan et al. [82] suggested an Optimal RSU Distribution Planner (ORDP) based on a Fusion Algorithm (FA) that relies on Evolutionary Genetic Algorithm (EGA) and D-Trimming. Here, d-trimming is a strategy that helps with reducing the number of RSUs proposed by the genetic algorithm depending on whether two routes can be served by a single RSU. ORDP takes into account the traffic density of a road segment, the budget, important road segments, a roadway’s accident history, and the transmission capabilities of an RSU. The scalability and efficiency of the planner are evaluated using simulated and realistic data sets, and it is discovered that ORDP outperforms alternative greedy techniques based on experiments focused on the city of Tamil Nadu, India. This framework allows the user to select the proper parameter configuration based on their needs that affect the decision of installing the RSU component, making the model viable and efficient. As limitations, this system does not include QoS parameters such as data transmission speed and delay.

We perform a qualitative comparison between the different approaches discussed above. Table 1 provides a comparative summary of the characteristics of various static deployment approaches. In Table 2, we summarize the static deployment strategies in terms of objectives, constraints, and model being applied.

### 4.2. Dynamic Deployment

The RSU deployment techniques that are based on a dynamic deployment are discussed in this subsection. Furthermore, instead of relying on expensive roadside infrastructure (such as RSUs), DSRC-equipped cars can serve as RSUs. In fact, vehicles utilized as temporary RSUs, parked cars used as RSUs, bus lines used as RSUs, and Unmanned Aerial Vehicles (UAVs) acting as RSUs are among the four sub-classes of RSU deployment techniques. It is worth noting that a lot of current research views dynamic deployment as a special technique for improving network connection. Furthermore, we do a qualitative evaluation of the various deployment options for dynamic RSUs. We perform a qualitative comparison between the different approaches discussed above.

#### 4.2.1. Vehicle Used as Temporary RSU

Ozan and Viriyasitavat in [88] proposed a new system known as a biologically inspired self-organizing network to allow some cars equipped with DSRC devices to be employed as temporary RSUs; the dynamic component of this placement technique is reflected by the dynamic selection of the cars. To provide a temporary RSU, a vehicle engaged in the collision (or a police car) can make a brief stop to execute the functions of a conventional RSU, such as disseminating security alerts to neighboring cars, where a gift-wrapping algorithm is proposed to meet these needs [89]. Such findings demonstrated that, unlike security messages, other forms of communications may be used. This approach is very useful, but it is limited by several assumptions. When the automobiles are too far away from one another, the communication link disconnects itself. Furthermore, the stops of ordinary cars (temporary RSUs) still leave a question mark on the system’s robustness and dependability.

#### 4.2.2. Parked Cars Used as RSUs

**Figure 5:**
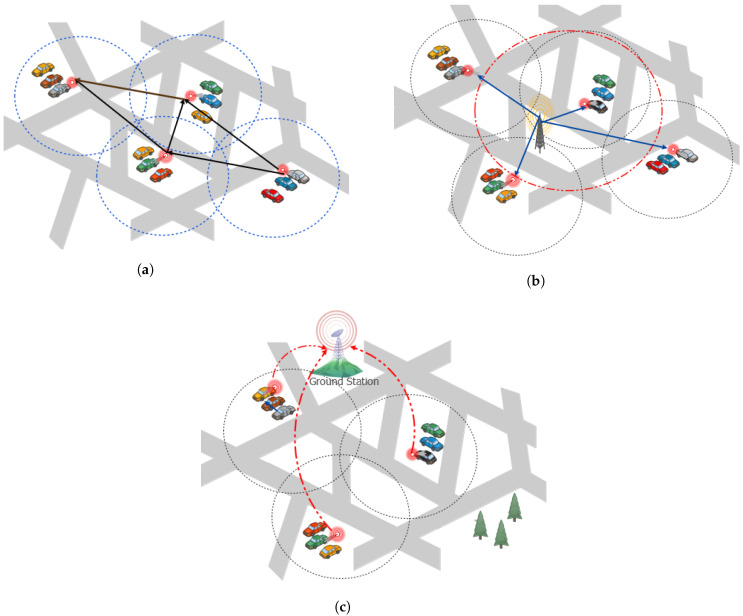
Modes of operation for parked cars acting as RSUs. (**a**) Parked cars form a mesh network with point-to-point links to other parked cars. (**b**) Parked cars extend the range of a fixed 802.11p RSU, acting as relays to it. (**c**) Parked cars with access to an uplink establish them selves as standalone RSUs.

The existence of large numbers of parked cars is a motivation to give those cars the role of RSUs using a self-organizing approach. This approach consists of three modes (Figure 5 summarizes these three modes). When there are no fixed RSUs in the urban area, parked cars create a network to support network connectivity to other moving vehicles (see Figure 5a). If there is a limited number of fixed RSUs in the area, parked cars in the vicinity of an RSU can act as relays to other nodes, extending the transmission range of the current fixed RSU (see Figure 5b). A parked car which is linked to a backbone uplink can leverage that link via the Internet, and thus establish itself as a standalone RSU (see Figure 5c).

In [90], its authors proposed a study with the aim to improve cooperative awareness and road traffic safety in urban areas using parked vehicles as relay nodes through two hop transmissions. To do this, each moving car sends out periodically beacon messages indicating its position and speed, which are picked up by parking nodes. A parked car will rebroadcast this beacon message as an RSU, allowing other moving cars to pick up the signal. This study compares message propagation using static RSUs, and shows that the number of RSUs has significantly decreased. Furthermore, moving vehicles can receive emergency signals sent by adjacent automobiles in a reasonable amount of time. This idea, however, requires more energy to be operational and does not solve coverage when an object occurs near a parked automobile.

In [91], the authors used parked cars as RSUs “Leveraging Parked Cars as Urban Self-Organizing Roadside Units”. This method introduced two operation modes for parked cars instead of an existing RSU and or standalone RSUs. The purpose of this idea is to increase safety applications in the event of an accident. In this case, an emergency message should be delivered to neighboring parked cars (nodes). After receiving this information, each node transmits a signal to its nearby vehicles, and so on. This information is used to split an urban area into equal cells (i.e., a cell map), and to determine which locations can be accessed by each vehicle. A decision algorithm is employed to determine if a parked automobile should become an RSU or go into power-saving mode (sleep). To validate this approach, the authors developed a realistic simulation platform integrating real maps, realistic vehicle mobility and traffic light patterns with mobility simulator, real building obstruction data, and empirical signal measurements. Results revealed that this strategy enhanced transmission coverage for safety applications even when only a limited number of parked automobiles were available. However, this algorithm requires only one-hop exchange between neighbor nodes to minimize the associated network overhead. In addition, the transmission coverage can be decreased if a mobile obstruction appears near an area parked. Indeed, a correction process is needed to oversee the decreased transmission range. We can note that the batteries in the parked cars do not recharge while the engine is turned off.

#### 4.2.3. Bus Line Management as RSU

**Figure 6:**
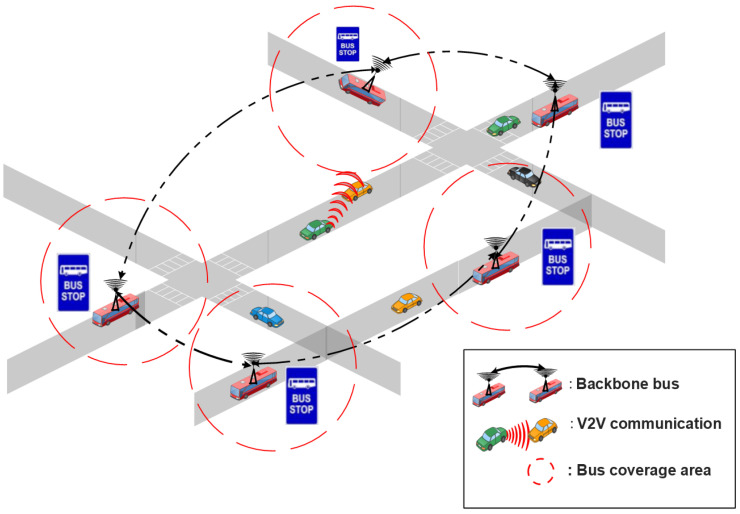
Mobile infrastructure based on backbone bus.

When there are no fixed RSUs existing in the urban area, the buses can constitute the backbone network, and can also play an important role in improving the messages dissemination process, as presented in Figure 6. Whenever there is a limited number of fixed RSUs, bus lines can be used as relay nodes to serve the data traffic between the vehicles and the existing RSUs.

Reis et al. in [92] developed a dynamic framework to enhance [91]. This type of solution is based on three modes of operation for parked cars in urban topologies. For all these modes, coverage maps will be generated for each individual car based on received signal power, dividing the urban areas into logical 2D cell maps. The authors designed and exploited DSRC radio signal strength measurements to evaluate obstacles and assure effective coverage by neighboring parked cars. In order to save energy in parked cars, the authors provide a dynamic decision procedure for determining whether a vehicle should become an RSU or enter a sleep mode. Simulation results revealed that a low number of parked cars in the urban area provided great connection coverage. Furthermore, the usage of such a relay system for a parking time of less than one day has no negative influence on the vehicle’s usefulness. Nerveless, regardless of the mode of operation (active/sleep), the parked automobiles are energy-constrained and can stop parking at any moment.

**Figure 7:**
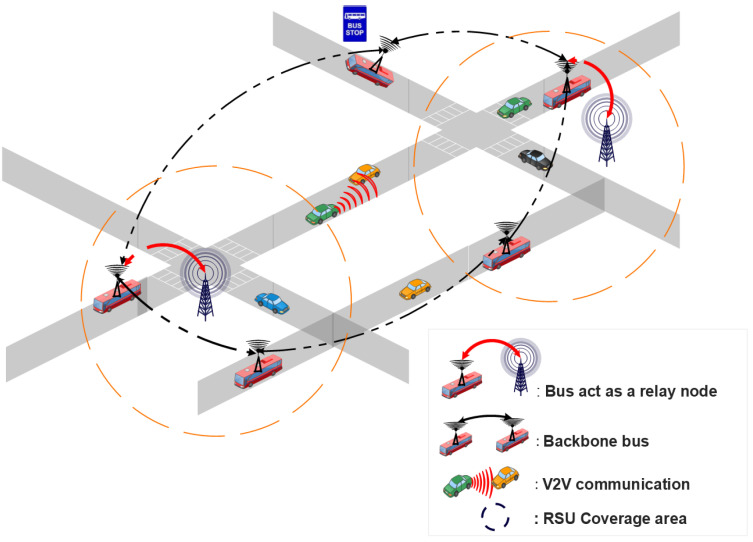
Mobile infrastructure based on VANET architecture.

Jiang and Du [93] proposed a two-tier architecture called BUS-VANET, based on high-tier and low-tier that leverages upon the predictable routes and timetables of buses. The high-tier comprises RSUs, Traffic Control Centers (TCCs), and bus routes. The automobiles equipped with DSRC devices, on the other hand, comprise the low-tier. When a low-tier node wishes to transmit a message, it must first register with a neighboring high-tier node in order to ascertain the delivery path given by the high-tier node. This architecture is summarized in Figure 7. The simulation results revealed that the two-tier BUS-VANET has the shortest delivery delay and the highest packet delivery ratio. Nonetheless, in the situation of a sparse road network, this technique did not take into consideration transmission services supplied by existing RSUs to provide the best QoS communications.

Kim et al. [94] suggested a new framework called the Budgeted Maximum Coverage Problem (BMCP) to optimize RSU deployment under a limited budget. Due to the high cost of a massive RSU deployment in wide metropolitan areas, this framework combines three different RSU deployment strategies: static, public mobile nodes (i.e., Buses) that are not controllable, and fully controllable mobile nodes (i.e., vehicles). The proposed approach consists of two steps, each of which uses a directed acyclic graph. For solving the maximum coverage problem, a greedy algorithm is applied in the first stage. To address the maximum coverage budget issue, the second stage similarly employs a greedy method. When compared to the situation of using a single deployment approach, the simulation results revealed that this framework provides a cost effective solution. As a limit, this study hypothesized that all mobile transportation does not suffer from any delay, and that the controllable mobile nodes do not suffer from traffic jams, which is not always the case in a realistic situation.

To achieve efficient vehicle communication in highway scenarios, Lee and Ahn [95] suggested an adaptive configuration strategy for placing mobile RSUs (mRSUs) in a backbone network in a cost effective manner. To formulate this problem, the authors use a binary linear programming model that considered the distribution of vehicles, wireless interference, and the speed of vehicles. This model facilitated the vehicle to access the RSUs with direct V2R communication, or with multi-hop relaying when the vehicles are outside the transmission range of the RSU. In addition, this model describes a process in which a roadside unit decides its state (active or inactive) based on the neighbor roadside unit and vehicles. Simulation results confirmed the performance of this model compared to hot-spot placement and uniform distribution of RSUs placement in terms of maximized cumulative throughput, cost effectiveness, and efficient placement. However, in an urban road environment, there are many more aspects to consider, such as direction, traffic signals, and so on, necessitating more careful mRSU management.

Heo et al. [96] show how static roadside units (sRSUs) can be replaced by buses used as mobile ones (mRSUs) in order to minimize the deployment and management costs and maximize the contact coverage between vehicles and roadside units using vehicle-to-infrastructure (V2I) communication. The performance trade-off and cost of using buses as mRSUs are addressed using mathematical analysis as well as real-world experiments that show that replacing static RSUs with mRSUs can maintain the same level of throughput, contact time, and inter-contact time as a function of replacement ratio. However, the scale of the experiment cannot match that of the simulation study due to practical constraints.

#### 4.2.4. Unmanned Aerial Vehicles Acting as RSUs

Unmanned Aerial Vehicles (UAVs), also known as drones, have recently experienced a significant adoption in the context of smart cities to overcome the limitations of communications between vehicles [97]. Several constraints have been acknowledged in VANETs, such as the limited number of RSUs, the high mobility of vehicles, existing obstacles, etc. These issues can be addressed by using UAVs as suitable candidates for improving the performance of vehicular networks [98]. Combining VANETs and UAVs also has advantages in terms of line-of-sight communication, load balancing, flexibility, and cost effectiveness. In particular, the UAVs can act as mobile RSUs and collect information from an area of interest, and transmit that information to vehicles, static RSUs, and other nearby UAVs [99]. In this case, there are several communication mechanisms available, including V2V, UAV-to-UAV (U2U), and Vehicle-to-UAV (V2U); such mechanisms rely on pure ad-hoc communications without any fixed infrastructure. Vehicle-to-Roadside-Unit (V2R) and UAV-to-roadside units (U2R) are established only when certain applications need to be run, such as Internet access.

Based on this concept, Oubbati et al. in [100] conceived a novel UAV-assisted reactive and flooding-based routing protocol that included a predictive technique to estimate the expiration time of discovered routing paths. It considers UAVs cooperating with road vehicles on an ad hoc manner to offer reliable routing paths. In this work, an algorithm that considers dynamic network topology was developed under the assumption that UAVs have complete knowledge of the device location. It has been investigated that optimal flight trajectories for UAVs can improve the ad hoc network connectivity. The experimental results of this research activity showed that the UAV-assisted VANET performed significantly better in terms of data delivery ratio and delay. However, the adopted discovery strategy may result in a high overhead, particularly in areas with a high vehicle density.

The same authors proposed a flooding technique in [101] that responds instantly to any network disconnection while avoiding existing obstacles. Indeed, a set of UAVs has been deployed to serve as backup solutions in the event that there is no connected routing path on the ground between the communicating nodes. Furthermore, the routing paths are established based on the longevity and regulation of each path, which are determined by the expiration time and the amount of traffic, respectively. Nonetheless, the UAVs’ adjustable mobility could be improved further to place them in the appropriate locations based on ground disconnections.

Cai et al. in [102] provided a cable-connected roadside unit (c-RSU) that can be combined with an unmanned aerial vehicle (UAV) assisted RSU (u-RSU), so that the latter can be dynamically changed to optimize coverage over time. Given a budget, the authors optimize the location of RSUs and u-RSUs based on the most Effective Traffic Coverage Ratio (ETCR). To tackle this problem, the authors formulate it as a knapsack problem of 0–1 integer programming, and offer a two layer improved greedy algorithm (TLIGA) that combines c-RSU and u-RSU deployment strategies: the first determines the location of c-RSUs, whilst the second determines the placement and number of u-RSUs. These methods are tested against other greedy algorithms on a road network. However, this proposal does not take into consideration realistic topologies where road complexity is present. In addition, the simulations’ results did not show the impact on QoS parameters. For u-RSU, energy requirement studies are needed.

As a summary, we performed a qualitative comparison between the different approaches discussed above. Table 3 provides a comparison of the characteristics of various dynamic deployment approaches. Table 4 presents a qualitative overview of dynamic deployment approaches in terms of objectives, constraints, and model being applied.

## 5. Open Issues and Future Research Directions

The deployment of an infrastructure is one of the most critical decisions when designing vehicular networks. RSU deployment is the task of defining the exact location of RSUs within the road network. In this article, we reviewed several works addressing the roadside unit deployment problem for vehicular ad hoc networks. Our goal is to summarize solutions proposed in the literature, to identify the limitations of present technologies, and to present research challenges as well as future research directions concerned in this research domain.

### 5.1. Realistic Deployment Strategy

On the topic of roadside unit placement, and the benefits these units bring to both overly sparse and overly dense networks, the existing body of work is now considerably mature [112]. Interesting work remains on how to best integrate these units into the existing networks. In addition, low vehicle density of sparse networks causes intermittent network connectivity and routing failures.

### 5.2. The Network Management as an RSU Deployment Constraint

In the years to come, the greatest challenge seems to be bringing Intelligent Transportation Systems (ITS) into the streets. To provide more profitable and efficient vehicular applications, the communication network management should enable easy establishment along roads and in low-density areas [113]. For this reason, the communication network management requires the governments to establish service level agreements for network providers. Since safety applications are time-critical (e.g., alert messages, warnings), and the decision-making is highly dependent on the data collected from the network, properly designing and managing the communication network is an essential step before deploying any fixed infrastructure.

### 5.3. Energy Saving

Roadside units are deployed along roadways where a direct connection to the electric grid is rarely available. In such cases, these roadside units will be equipped with rechargeable batteries, necessitating expensive human involvement for upkeep [114]. As a result, effective roadside unit operation techniques are required to reduce energy use. Energy harvesting appears to be a viable option for powering and charging nodal batteries in vehicular networking. Furthermore, taking into account the specific characteristics of the highly mobile vehicle network, the viability of energy harvesting technologies in a vehicular context must be studied. To this aim, an energy and communication-driven model for IoV scenarios is presented in [115], in which roadside units (RSUs) must be assigned and reassigned to operating vehicles on a regular basis.

### 5.4. Dynamic Vehicle Mobility

A thorough knowledge of the urban and rural vehicle mobility is certainly a crucial aspect for the deployment of roadside units [116]. In effect, any RSU deployment strategy should consider strong and active models of vehicle mobility to develop and validate more realistic RSU placement in terms of mathematics formulations, optimization models, and algorithms. With the advent of fifth communication networks and self-driving cars [117], many academic efforts are geared toward more efficient mobility management solutions [118].

### 5.5. Data Security

Because of the open deployment environment, network traffic between all RSUs could be intercepted by potential network attacks, resulting in a degraded user experience, disrupted RSU workload scheduling, and even data leakage [119]. To prevent these attacks, the personal data such as location or speed, which requires anonymity and protection of drivers’ privacy, the data security is a major concern in RSU deployment. Indeed, the roadside units play a key role in the security framework; to this end, they can assume the responsibility of delivering pseudonyms to cars that enter its transmission range [120]. The RSUs undergo a shuffling process periodically by exchanging sets of pseudonyms with each other, hence allowing for the reuse of pseudonyms, but not by the same car. Tackling this concern may maximize anonymity when vehicles communicate with other nodes in the network through the use of robust security solutions based on an efficient RSU placement.

### 5.6. Communication Architecture

The discussions on Intelligent Transportation Systems, as well as the insights into the application of the new concept of Internet of Vehicles (IoV) [121], lead researchers to propose a novel architecture of vehicular communications [122]. A communication architecture based on (IoV) not only includes vehicles and RSUs, but also other communication devices: the cellular networks infrastructure, personal devices, and sensors [123]. Consider that this new communication architecture is a key challenge for a new deployment of a heterogeneous infrastructure of vehicular networks that should take into account the new transmission modes, namely vehicles to sensors, vehicles to pedestrian, and vehicle to cloud/fog computing.

### 5.7. Heterogeneous Connectivity

The dynamicity of the vehicles in urban environments presents itself as a feature and challenge that allows data propagation and heterogeneous connectivity with different network technologies [124]. These networks differ from traditional networks in many ways. The first difference lies in the nature of the nodes that form them, such as automobiles, trucks, buses, and taxis, as well as equipment attached to roads where they all have wireless communication interfaces. In addition, these nodes have high mobility, and their trajectory follows the limits and direction defined by public pathways [125].

### 5.8. RSUs and Edge Server Deployment

RSUs may be seen as edge servers in the context of the Internet of Vehicles [126], helping to gather information submitted by vehicles, and assisting with information transfer. The ideal edge server deployment technique, to cover as many vehicle nodes as feasible in order to meet the coverage and connectivity of IoVs, is hence a research problem [127]. Furthermore, the quantity and position of available RSUs in the network affects the time delay of transmission in IoV, which is a difficulty in and of itself.

## 6. Conclusions

Future intelligent transportation systems (ITS) which address important issues such as traffic safety and efficiency, as well as comfort services, will rely heavily on vehicular networking. Maintaining network transmission coverage is one of the most actively studied issues related to the Internet of Vehicles (IoV). The dynamic topology of IoV, on the other hand, is defined by the fast speed of the vehicles, and the availability of alternative routes. In order to address the coverage issue, RSU deployment is a primary solution that allows the IoV to maintain a strong connectivity. However, the RSU deployment is mainly influenced by factors such as vehicle mobility (density, speed), vehicles’ location, complex roadways, routing protocols, and QoS settings. This paper reviewed the RSU deployment in IoV, summarizing and analyzing the most proposed approaches in the literature by examining the achieved results and their evaluation methods. Depending on the mobility of vehicles, and the strategy for deploying RSUs in the target geographic areas, we classified the state of the art of the RSU placement strategies into two main categories, namely static and dynamic deployment. In addition, a comprehensive taxonomy of RSU-based IoV deployment was provided, including transmission technologies, deployment objectives, network metrics, deployment challenges, etc. As future work, to achieve a more efficient network through an efficient deployment strategy which integrates new applications, we expect to develop a novel RSU deployment scheme based on the idea of the hybridization of both static and dynamic schemes.

## Figures and Tables

**Table 1:** A comparison between the various static deployment approaches.

Sub-Class	Ref	Typologies				Communication				RSUs Locations			
		Highway	Urban Complex	Urban Grid	Rural	V2V	V2R	Muti-Hop	Backbone network	Intersection	Road Segment	Uniform Distribution	A Distinct Locations
Analytic Study	[60]	✓				✓	✓		✓			✓	
	[44]	✓					✓	✓			✓		
	[45]			✓			✓			✓			
	[61]										✓		
	[62]										✓		
Geometry Parameters	[43]			✓		✓	✓						✓
	[64]		✓				✓						
	[65]		✓				✓		✓				✓
	[67]		✓				✓		✓				✓
Transmission Time	[15]			✓			✓			✓	✓		
	[50]			✓		✓	✓	✓		✓			
	[68]	✓					✓					✓	
	[69]		✓	✓	✓		✓			✓			
	[70]			✓			✓	✓		✓			
	[71]	✓				✓	✓	✓			✓		
	[72]	✓					✓						✓
Maximum coverage	[53]		✓				✓			✓			
	[73]			✓			✓	✓		✓			
	[75]			✓			✓			✓			
	[76]		✓	✓	✓	✓	✓			✓			
	[77]		✓				✓			✓			
	[79]		✓				✓			✓			
	[80]			✓			✓			✓			
Network Area Density	[32]		✓			✓	✓		✓	✓			
	[78]		✓	✓			✓			✓			
	[81]		✓			✓	✓						✓
	[82]	✓	✓				✓				✓		

**Table 2:** A qualitative overview of static deployment approaches.

Ref	Main Objective	Constraints	Model	Algorithm	Compared to	Mobility Trace	Simulator
[60]	Maximize the deployment	Connectivity probability	Mathematics study	Randomized	Optimal algorithm [83]	100 km highway	Specific
	distance	threshold *p* and the time *t*				segment	
[44]	Maximize the achievable	Deployment budget	ILP	Capacity Maximization	Uniformly distributed	1250 m by 150	VanetMobisim, ns-2
	throughput in the network to			Placement (CMP) Strategy	and hotspot placements	highway	
	aggregate direct and multi-hop						
	communication						
[45]	Minimize the reporting time	The RSUs number	ILP	BIP and BEH	Between them	Manhattan topology	Specific
[61]	Maximize the coverage	*n* RSUs number	(D1RD)	OptGreDyn, Greedy2P3	OptAll, OptDynLim, BEP [45],	No mobility trace	MATLAB
				and Greedy2P3E	GreedyMiddle [84]		
[62]	Maximize the coverage	*n* RSUs number	(D1RD)	OptDynLim	OptAll and Genetic	No mobility trace	MATLAB
[43]	Maximise the RSU range	Required QoS	Voronoi graph	Voronoi diagram	Uniform distribution	Nashville, TN, USA	SUMO, ns-2
[64]	Maximize the coverage.	Budget sparse coverage	Geomantic	α-DBSCAN,	α-coverage [85]	Ottawa’s downtown	SUMO, ns-2
	Minimize the cost	Qualified sparse coverage	and ILP models	genetic and greedy			
[65]	Minimize the delay	The RSUs number	CDT	Constrained Delaunay	GeoCover [64] and	Ottawa’s downtown,	EXataCyber-5.4
					α-coverage [85]	Manhattan, and Rome	
[67]	Maximize the coverage	Time required for	Geometric model	genetic	Geographic and	Madrid, Valencia	SUMO
		emergency messages			D-RSU [81]	(Spain)	
[15]	Maximize the coverage	Delay-bounded	0–1 variation Knapsack	binary differential evolution	Genetic (BMCP-g)	Zhengzhou, China	SUMO
	of road segments	and cost-limited	problem (DBCL)				
[50]	Maximize the benefit of serving	The expected delivery	FLP	ILP-based clustering	Greedy and ILP	Manhattan grid	MATLAB
	the data dissemination tasks	requirement					
[68]	Minimize the cost	Delay bound of transmitting	Clustering model	Mathematical study	No comparison	No real topology	Specific
		alert messages				area	
[69]	Maximize the coverage and	The RSU number	MCTTP	Greedy and Genetic	Between them	Zurich traces [86]	Specific
	minimize dissemination time						
[70]	Minimize dissemination time	Coverage radius	ILP	Safety-Based RSU	Mesh deployment policy	Chicago, IL, USA	SUMO, ns-2
				Placement (S-BRP)			
[71]	Minimize the network latency due	The deployment budget	Delay Minimization	ILP	Cost-effective strategy	No realistic trace	VanetMobisim, ns-2
	to direct and multi-hop connections		Problem		and uniform distribution		
[72]	Maximize the interconnection gap	The contact time	Gamma deployment	Greedy and	The densest locations	Cologne, Germany [87]	SUMO
		threshold	strategy	hill climbing			
[53]	Maximizing coverage and connectivity	Minimal number	Multi-objective	Genetic	Greedy	Manhattan topology	Specific
	of vehicles contacting the RSU	of RSUs					
[73]	Minimize the RSUs number	Required QoS	SCP	Greedy	Uniform and	Manhattan topology	Specific
		data delivery			Random placement		
[75]	Maximize the number of distinct	The RSUs number	MCP	(PMCP-b)	MCP-kp and MCP-g [47]	Cologne, Germany	SUMO
	vehicles contacting the infrastructure						
[76]	Maximize the number of vehicles	Time overhead for vehicles	MCTTP	Genetic	Greedy	Cologne and Zurich	Specific
	connected to a subset of RSUs	to connect RSUs					
[77]	Maximize coverage	Minimum number of RSUs	VCP	AC-RDV	Genetic, Greedy and HGA	No realistic trace	Specific
[79]	Maximize coverage	No constraints	Multi-objective	(MODE-deg)	NSGA-II, MOEA/D,	Random graphs	Specific
	Minimize the cost				and MOEA/D-arg		
[80]	Maximize vehicles-access	Limited number of RSU	Powerful RSU	Genetic	BEH heuristic [45]	Dalian city, China	Specific
	demands to RSU		deployment Model				
[32]	Maximizing the travel time	Cost-limited	Aggregation scheme	Genetic	Uniform distribution	Brunswick, Germany	VISSIM, ns-2
	savings of cars				Strategy		
[78]	Maximize coverage and	Overlapped area	Intersection priority	Greedy, dynamic		Seoul, South Korea.	SUMO, ns-2
	minimize the RSUs number			and hybrid	between them		
[81]	Minimize the safety message time	Deployment cost	Mobility model	D-RSU approach	Uniform Mesh deployment	Madrid, Spain	SUMO, ns-2
[82]	Finding optimal location for RSUS	Installation budget.	Optimal RSU distribution	Genetic and	Greedy	Tamil Nadu, India	VISSIM
		Transmission rang of RSUs	planer (ORDP)	D-Trimming			

**Table 3:** Comparison between the various dynamic deployment approaches.

Sub-Class	Ref	Typologies			Communication				RSUs Locations				
		Highway	Urban Complex	Urban Grid	V2V	V2R	U2U	Backbone Network	Vehicles as RSUs	Bus as RSU	Parked Cars	Fixed RSUs “Intersection”	UAV Acting as RSUs
Vehicle used as temporary RSU	[88]		✓		✓				✓				
Parked cars as RSU	[90]			✓	✓						✓		
	[91]		✓		✓	✓					✓		
	[92]		✓		✓						✓	✓	
Bus line management as RSU	[93]		✓		✓	✓		✓		✓			
	[94]		✓		✓	✓		✓	✓	✓		✓	
	[95]		✓			✓		✓		✓			
	[96]			✓	✓	✓		✓		✓		✓	
UAV acting as RSUs	[100]		✓		✓		✓	✓				✓	✓
	[101]		✓		✓		✓	✓				✓	✓
	[102]			✓	✓	✓		✓				✓	✓

**Table 4:** Qualitative overview of dynamic deployment approaches.

Ref	Main Objective	Constraints	Model	Algorithm	Compared to	Mobility Trace	Simulator
[88]	Maximize the network connectivity	Boundary of the network	Biologically inspired	Distributed gift-wrapping [103]	Standard scheme	CA-based mobility	Specific
		coverage polygon	Self-organizing network			model [104]	
[90]	Maximize the coverage area	Upper bound for		A relaying algorithm	Static deployment	Manhattan Grid and	Veins [105]
	and signal attenuation	safety message				Ingolstadt, Germany	
[91]	Maximize the coverage of	Only 1-hop exchange	Self-organizing	Decision algorithm	Reference optimal	Porto, Portugal	SUMO
	parked cars network	of coverage maps	network approach		scenarios		
[92]	Maximize the coverage of the	Limited number of parked	Self-organizing	On-line, greedy	Scenario without RSUs	Porto, Portugal	SUMO
	parked network of parked cars	cars	network approach				
[93]	Minimize the number of switches	Limitation of package	BUS-VANET	Longest registration	Random and shortest	Minneapolis, USA	SUMO, ns-3
	from vehicles to high-tier nodes	delivery delay	architecture		distance selection		
[94]	Maximize the spatio-temporal	Limited deployment budget	Budgeted maximum	α-approximation	Single deployment strategy	San Francisco, USA	SUMO
	coverage		coverage problem (BMCP)	algorithm	(only static or mobile)		
[95]	Minimize the mRSU number in	Maximum capacity of each	Adaptive mRSU	Binary linear programming	All RSUs in active state	No real topology area	Veins
	active state (ON-state)	mRSU	configuration mechanism	algorithm	(only static or mobile)		
[96]	Optimize the performance network	The replacement cost of sRSUs	Mathematical analysis	No algorithm	With and without mRSUs	City of Manhattan	SUMO, ns-3
	in terms of throughput, contact time,	needs through mRSU					
	and inter-contact time						
[100]	Optimizing VANET	Coverage area of UAVs	Routing process based	UAV-assisted	RBVT-R [106], OLSR [107],	Manhattan grid	SUMO, ns-2
	routing process	and existing obstructions	on flooding technique	routing protocol	CRUV [108], and UVAR [109]		
[101]	Maximizing the number of	Coverage area of UAVs	UAV-assisted reactive	U2RV routing protocol	CRUV [108], and UVAR [109]	Zurich, Switzerland	SUMO, MobiSim
	alternative solutions, and	and existing obstructions	routing protocol		MURU [110], and AGP [111]		
	thus the delivery ratio						
[102]	Maximal effective traffic	Given tough budget bound	Knapsack problem	Greedy a (TLIGA)	Random-c, Greedy-c and Greedy-u	Grid topology	Specific
	coverage ratio (ETCR)			algorithm			

## Data Availability

Not applicable.

## Abbreviations

The following abbreviations are used in this manuscript:

MANET	Mobile Ad-hoc Network
UAV	Unmanned Aerial Vehicle
VANET	Vehicular Ad-hoc Network
GPS	Global Position System
OBU	On-Board Units
RSU	Roadside Units
ITS	Intelligent Transport System
IoV	Internet of Vehicles
DSRC	Dedicated Short Range Communication
V2V	Vehicle-to-Vehicle communication
V2R	Vehicle-to-roadside units
I2I	Infrastructure-to-Infrastructure communication
QoS	Quality of Service
SCP	Set Coverage Problem
MCP	Maximum coverage problem
FLP	Facility Location problem
VCP	Vertex Coverage Problem
ILP	Integer Linear Programming
BEH	Balloon Expansion Heuristic
D1RD	One-Dimensional RSU deployment problem
CDT	Constrained Delaunay Triangulation
MCTTP	Maximum Coverage with Time Threshold Problem
HGA	Heuristic Genetic Algorithm
SUMO	Simulation of Urban MObility [128]
ns-2 and ns-3	Network Simulator, versions 2 and 3 [129,130]
VISSIM	in German “Verkehr In Städten—SIMulationsmodell” [131]
